# Engagement of CEACAM1 by *Helicobacter pylori* HopQ Is Important for the Activation of Non-Canonical NF-κB in Gastric Epithelial Cells

**DOI:** 10.3390/microorganisms9081748

**Published:** 2021-08-16

**Authors:** Karin Taxauer, Youssef Hamway, Anna Ralser, Alisa Dietl, Karin Mink, Michael Vieth, Bernhard B. Singer, Markus Gerhard, Raquel Mejías-Luque

**Affiliations:** 1 Institute for Medical Microbiology, Immunology and Hygiene, Technical University Munich, 81675 Munich, Germany; karin.taxauer@tum.de (K.T.); youssef.hamway@tum.de (Y.H.); anna.ralser@tum.de (A.R.); alisa.dietl@tum.de (A.D.); karin.mink@tum.de (K.M.); markus.gerhard@tum.de (M.G.); 2 Institute of Pathology, Friedrich-Alexander University Erlangen-Nuremberg, Klinikum Bayreuth, 95445 Bayreuth, Germany; vieth.lkpathol@uni-bayreuth.de; 3 Institute of Anatomy, Medical Faculty, University of Duisburg-Essen, 45147 Essen, Germany; BBSINGER@gmx.de

**Keywords:** non-canonical NF-ĸB, NIK, p52, *H. pylori*, HopQ, CEACAM, gastric cancer, human gastric primary cells

## Abstract

The gastric pathogen *Helicobacter pylori* infects half of the world’s population and is a major risk factor for gastric cancer development. In order to attach to human gastric epithelial cells and inject the oncoprotein CagA into host cells, *H. pylori* utilizes the outer membrane protein HopQ that binds to the cell surface protein CEACAM, which can be expressed on the gastric mucosa. Once bound, *H. pylori* activates a number of signaling pathways, including canonical and non-canonical NF-κB. We investigated whether HopQ–CEACAM interaction is involved in activating the non-canonical NF-κB signaling pathway. Different gastric cancer cells were infected with the *H. pylori* wild type, or HopQ mutant strains, and the activation of non-canonical NF-κB was related to CEACAM expression levels. The correlation between CEACAM levels and the activation of non-canonical NF-κB was confirmed in human gastric tissue samples. Taken together, our findings show that the HopQ–CEACAM interaction is important for activation of the non-canonical NF-κB pathway in gastric epithelial cells.

## 1. Introduction

*Helicobacter pylori* colonizes the gastric mucosa of every second human being worldwide [1]. Although the majority of infected individuals are asymptomatic, *H. pylori* infection can result in peptic ulcer, gastric adenocarcinoma and mucosa-associated lymphoid tissue (MALT) lymphoma [2,3]. MALT lymphoma arises from long-term *H. pylori* infection resulting in the growth of malignant lymphatic cell clones [4,5,6]. Early-stage gastric MALT lymphomas are antigen-dependent illnesses and hence, *H. pylori* eradication therapy using antibiotics induces regression in three out of four patients [5]. In contrast, late-stage MALT lymphomas show high-grade transformation and acquire chromosomal translocations becoming antigen-independent and resistant to the eradication therapy [7]. In addition, *H. pylori* is one of the major risk factors for developing gastric cancer, classified as a class I carcinogen by the WHO in 1994 [8,9,10,11]. In 2018, gastric cancer accounted for about one million new cancer cases worldwide and was the fifth most common cancer type [12]. Intestinal-type gastric cancer develops over decades through a multistep process initiated by infection-induced gastritis, that further progresses to atrophic gastritis, intestinal metaplasia, dysplasia, and cancer [13,14], while no precursor lesions have been identified for diffuse tumours.

To colonize the gastric epithelium, *H. pylori* initially has to adhere to gastric epithelial cells (reviewed in [15]). The bacterium achieves this by expressing several outer membrane proteins (OMP) that interact with different host receptors [16,17,18]. In previous work, the adhesin HopQ was found to bind to human carcinoembryonic antigen-related cell adhesion molecules (CEACAMs), mainly CEACAM1 and 5, and to a lesser extent to CEACAM3 or 6 [19,20,21]. Importantly, we showed that HopQ-CEACAM interaction is essential for type IV secretion system (T4SS)-dependent CagA translocation and interleukin 8 (IL8) secretion [19]. Inside the host cell, CagA interacts with signalling molecules such as Src and c-Abl kinases, resulting in its phosphorylation [22]. This affects a number of host signalling pathways that regulate the expression of cytokines and growth factors involved in immune responses, cytoskeletal rearrangements, and cell elongation [23,24,25]. One of those signalling pathways is the nuclear factor-κB (NF-κB). However, whether activation of NF-κB is completely CagA-dependent, T4SS dependent, or just *H. pylori* strain-specific, is still under debate [26,27,28,29].

The NF-κB pathway is divided into two signalling arms, the canonical and the non-canonical pathway, according to the IκB kinase (Iκκ) subunits employed [30,31]. Upon *H. pylori* infection, the canonical NF-κB pathway is activated in gastric epithelial cells or infiltrating immune cells [28,29,30]. Accumulating evidence also indicates an important role for non-canonical NF-κB in *H. pylori* induced gastric malignancies [32,33,34]. The non-canonical NF-κB pathway is activated by a defined subset of tumour necrosis factor receptor (TNFR) superfamily members such as lymphotoxin β receptor (LTβR), B cell activating factor belonging to TNF family receptor (BAFFR), CD40, receptor activator for NF-κB (RANK), TNF-related weak inducer of apoptosis (TWEAK), TNFR2 and other TNFR superfamily members [30,35]. LTβR, which is expressed on lymphoid stromal and epithelial cells, is activated by two main ligands LTα_1_β_2_ or LIGHT (homologous to lymphotoxins exhibits inducible expression, competes with herpes simplex virus glycoprotein D for the receptor HVEM expressed on T lymphocytes) [35]. Receptor activation leads to the stabilization of NF-κB inducing kinase (NIK) that phosphorylates the Iκκα homodimer resulting in the phosphorylation of p100 and degradation of p100 to p52, which then forms a heterodimer with RelB [36]. The formed heterodimer translocates into the nucleus to induce gene expression [35]. Previous work in our laboratory demonstrated that activation of the non-canonical NF-κB pathway via lymphotoxin β receptor (LTβR) signalling aggravates the pro-inflammatory response to *H. pylori* in gastric epithelial cells, while blocking LTβR signalling reduces gastric inflammation. Activation of this pathway was mostly induced by LTβ and depended on the presence of a functional T4SS in the bacterium [32].

Considering that HopQ-CEACAM binding is essential for T4SS functionality [33,37,38,39], we sought to investigate whether HopQ-CEACAM interaction influences the activation of non-canonical NF-κB signalling in gastric epithelial cells. Our results indicate that HopQ-CEACAM interaction not only effects CagA translocation or IL8 secretion, but also influences the activation of the non-canonical NF-ĸB pathway.

## 2. Materials and Methods

### 2.1. Cell Culture

Gastric cancer cell lines: AGS (ATCC CRL-1739), KatoIII (ATCC HTB-103), MKN7 (JCRB1025), MKN45 (JCRB0254), NCI-N87 (ATCC CRL-5822), NUGC-4 (JCRB0834), SNU1 (ATCC CRL-5971), St2957 (CVCL_9557) and St3051 (CVCL_9558) were cultured in Dulbecco’s modified Eagle’s medium (DMEM) and supplemented with 10 % (*v*/*v*) FCS and 1% (*v*/*v*) Pen/Strep. Chinese hamster ovary cells (CHO) (ATCC CCL-61) and vector control CHO-CEACAM1-4L (CHO CC1) [21] were cultured in DMEM and supplemented with 10 % (*v*/*v*) FCS and 1% (*v*/*v*) Pen/Strep. Cells were kept in a humidified atmosphere at 37 °C and 5% (*v*/*v*) CO_2_. Regular mycoplasma testing was performed.

### 2.2. H. pylori Strains

P12 [40] and G27 [41] *H. pylori* strains were grown on Wilkins-Chalgren (WC) blood agar plates supplemented with Dent (OXOID, Hampshire, UK) and kept at microaerophilic conditions at 37 °C and 10% (*v*/*v*) CO_2_. G27/P12 isogenic strains, G27Δ*hopQ* [19], G27Δ*cagE* [42], P12Δ*hopQ* [20], P12Δ*cagA* [43] and P12Δ*cagE* [44], were grown in plates containing 50 µg/mL kanamycin, 15 µg/mL of chloramphenicol or 50 µg/mL streptomycin under the same conditions.

### 2.3. Infection Experiments

Cells were seeded at a density of 1 × 10^5^ in a 24-well plate (for protein and ELISA) or 5 × 10^5^ in a 6-well plate (for RNA). The next day, the cell medium was replaced by DMEM containing only FCS without antibiotics. On the day of infection, cells of one well were counted to calculate the number of bacteria needed. *H. pylori* was grown for 48 h on WC agar plates with Dent supplement and collected in BHI/10% FCS medium. Bacterial density was determined by measuring the optical density (OD600), with OD_600_ = 1 = 2 × 10^8^ bacteria. *H. pylori* was applied at a multiplicity of infection (MOI) of 10, 20, or 50 and incubated for eight hours. After the incubation time, the supernatant was collected to perform IL8 ELISA, and cells were washed once with 1x PBS and lysed for protein or RNA expression analysis.

### 2.4. Binding Assay

*H. pylori* was grown on WC Dent agar plates for 48 h, the optical density was measured and adjusted to 2 × 10^8^ bacteria in 1 mL media, and the bacteria was stained for 30 min with 10 µM carboxyfluorescein succinimidyl ester (CFSE, eBioscience, San Diego, CA, USA) [39]. The labeled bacteria were washed twice and added at an MOI of 10 to 1 × 10^5^ cells, which were previously seeded in a 96-well V bottom plate. After an incubation period of 30 min under constant shaking, the supernatant was discarded, and the infected cells were washed 4 times with PBS. After fixing cells in 0.5% (*v*/*v*) PFA/PBS, the cells were analysed on a CytoFLEX S flow cytometer (Beckman Coulter, Brea, CA, USA). Cells not incubated with labeled bacteria served as a negative control. Binding was assessed, after pre-gating on single cells, based on fluorescent signals using FlowJo software (version 10.7.2).

### 2.5. Immunofluorescence (IF)

To visualize the binding of *H. pylori* to gastric cells, 1 × 10^5^ MKN45 and NUGC-4 gastric cancer cells were seeded on coverslips and grown for 24 h. CFSE-labeled wild type *H. pylori*, or isogenic mutant strains, were added at an MOI of 10 and co-incubated with the cells for 3 h. Infected cells were fixed with methanol/acetone, stained with phalloidin (red, actin), and mounted with DAPI-containing media (Vector Labs, Burlingame, CA, USA). Images were taken using an SP5 confocal microscope (Leica, Wetzlar, Germany).

### 2.6. Immunohistochemistry (IHC)

Human gastric biopsy samples from paraffin-embedded blocks were obtained from the tissue bank of the Institute of Pathology, Klinikum Bayreuth in Germany, after the approval of the local ethics committee (155_20B). Briefly, sections were stained for NF-κB 2 (#3017, Cell Signaling Technology, Danvers, MA, USA; 1:800) or CEACAM1 (provided by B. B. Singer, Essen, Germany; 5 µg/mL). Head-induced antigen retrieval was conducted using 0.01 mol/L sodium citrate (pH 6). The primary antibody was incubated at 4 °C overnight and the bound secondary antibody (Promega, Madison, WI, USA) was detected using diaminobenzidine solution (DAB). Slides were scanned and examined using an Olympus Virtual Slide Imaging System (Olympus, Shinjuku, Tokio, Japan).

### 2.7. Enzyme-Linked Immunosorbent Assay (ELISA)

To test for IL8 concentration in culture supernatants, samples were diluted at 1:40 (MKN45) or 1:10 (NUGC-4) in culture media or were used pure (SNU1). ELISA was performed according to the manufacturer’s instructions (Ready-Set Go ELISA Kit, Invitrogen, Carlsbad, CA, USA).

### 2.8. Western Blot

To check for protein expression, equal volumes of protein lysate were loaded on an 8% or 6% sodium dodecyl sulphate–polyacrylamide gel, and electrophoresis (SDS page) was performed. Separated proteins were transferred onto a nitrocellulose membrane (Amersham Protran 0.45, GE Healthcare, Chicago, IL, USA). The membrane was blocked with 5% (*w*/*v*) non-fat milk in TBS-T buffer (Tris-buffered saline supplemented with 0.1% Tween 20 (*v*/*v*)) at room temperature for one hour. The blocked membrane was probed with primary antibodies targeting NF-κB2 (#3017, Cell Signaling Technology; 1:1000), and CEACAM1 + 5 (provided by B. B. Singer; 2.5 μg/mL) at 4 °C overnight. GAPDH was used as a protein loading control (#2118, Cell Signaling Technology; 1:1000). After washing, the membrane was incubated with secondary HRP conjugated anti-rabbit IgG or anti-mouse IgG antibody (Promega). Proteins were detected by applying ECL Western blotting detection reagent (Thermo Fisher Scientific, Waltham, MA, USA) and quantified with LabImage software (INTAS, Ahmedabad, India). Protein levels were normalized to GAPDH.

### 2.9. Quantitative Real-Time PCR (qPCR)

RNA was isolated using an RNA Miniprep Kit (Sigma, St. Louis, MO, USA). Next, DNase treatment was performed, and 1 µg RNA was used to prepare cDNA. *LTβ*, *A20*, *CEACAM1*, *CEACAM5*, *CEACAM6*, *CCL2*, *CXCL10*, and *GAPDH* primer sequences are shown in Table 1. Relative mRNA levels were calculated using the equation 2^−∆ct^ after normalizing values to the housekeeping gene *GAPDH*. Fold mRNA expression was calculated using the 2^−∆∆ct^ formula. Thus, values were first normalized to the housekeeping gene and afterwards to the values of the control samples.

### 2.10. Statistics

One-way analysis of variance (ANOVA) with Bonferroni’s correction for multiple comparisons was used for normally distributed data. For not normally distributed data, the ANOVA Kruskal–Wallis with Dunn’s multiple comparisons test were performed. Statistical significance was determined as * *p* ≤ 0.05; ** *p* ≤ 0.01; *** *p* ≤ 0.001; **** *p* ≤ 0.0001.

## 3. Results

### 3.1. H. pylori HopQ Is Involved in the Activation of the Non-Canonical NF-κB Pathway

**Figure 1:**
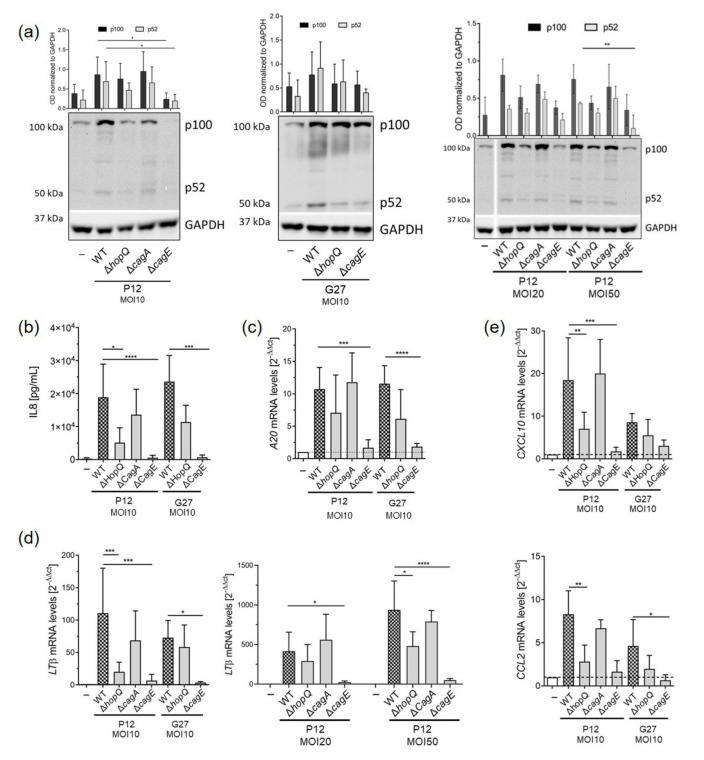
Activation of the non-canonical NF-κB pathway upon *H. pylori* infection in MKN45 gastric cells. MKN45 cells were infected with P12 or G27 wild type *H. pylori* strains or isogenic mutant strains at an MOI of 10, 20 or 50 for 8 h. (**a**) p100 to p52 processing detected by Western blot. GAPDH was used as loading control. (**b**) IL8 secretion was measured by ELISA. (**c**) *A20*, (**d**) *LTβ* and (**e**) *CXCL10* and *CCL2* mRNA levels were analyzed by qPCR. *GAPDH* was used as a housekeeping gene. All qPCR values were normalized to uninfected controls and calculated using the 2^-ΔΔct^ equation. Graphs show mean ± SD of at least three independent experiments. One-way ANOVA with Bonferroni correction (**a**,**c**–**e**) or Kruskal–Wallis with Dunn’s correction (**b**). * *p* ≤ 0.05; ** *p* ≤ 0.01; *** *p* ≤ 0.001; **** *p* ≤ 0.0001.

We previously observed that the activation of non-canonical NF-κB in response to *H. pylori* infection occurred on a secondary loop post-canonical NF-κB activation that required a functional T4SS [32]. Since HopQ is important for proper T4SS functionality, CagA translocation and NF-κB activation, we explored whether HopQ plays a role in the activation of the non-canonical NF-κB pathway. Thus, we infected MKN45 gastric cancer cells with wild type *H. pylori* strains as well as isogenic HopQ deficient strains. CagA as well as CagE knockout strains served as controls. Wild type *H. pylori* infection resulted in the activation of non-canonical NF-κB, as p100 was efficiently processed to p52, regardless of the strain used for infection (Figure 1a). In contrast, only reduced or no processing of p100 was detected in cells infected with HopQ or CagE deficient strains, while lack of CagA did not affect activation of the pathway (Figure 1a), as observed previously [32]. As expected, activation of canonical NF-κB was also hampered after infection with P12Δ*hopQ*, as reduced levels of IL8 secretion (Figure 1b) as well as mRNA levels of *A20* (Figure 1c) were observed. Likewise, the expression of *LTβ*, a ligand which is crucial for activation of the non-canonical NF-κB pathway upon *H. pylori* infection, was lower in MKN45 gastric cancer cells infected with HopQ mutant strains compared to expression in cells infected with wild type bacteria (Figure 1d). In addition, the expression of NF-κB target genes *CXCL10* and *CCL2* was reduced when HopQ was missing (Figure 1e). Similar effects were observed at a higher MOI (MOI 20 or 50) in infected MKN45 cells (Figure 1a,d).

### 3.2. Gastric Cells Express Different Levels of CEACAMs That Are Altered upon H. pylori Infection

**Figure 2:**
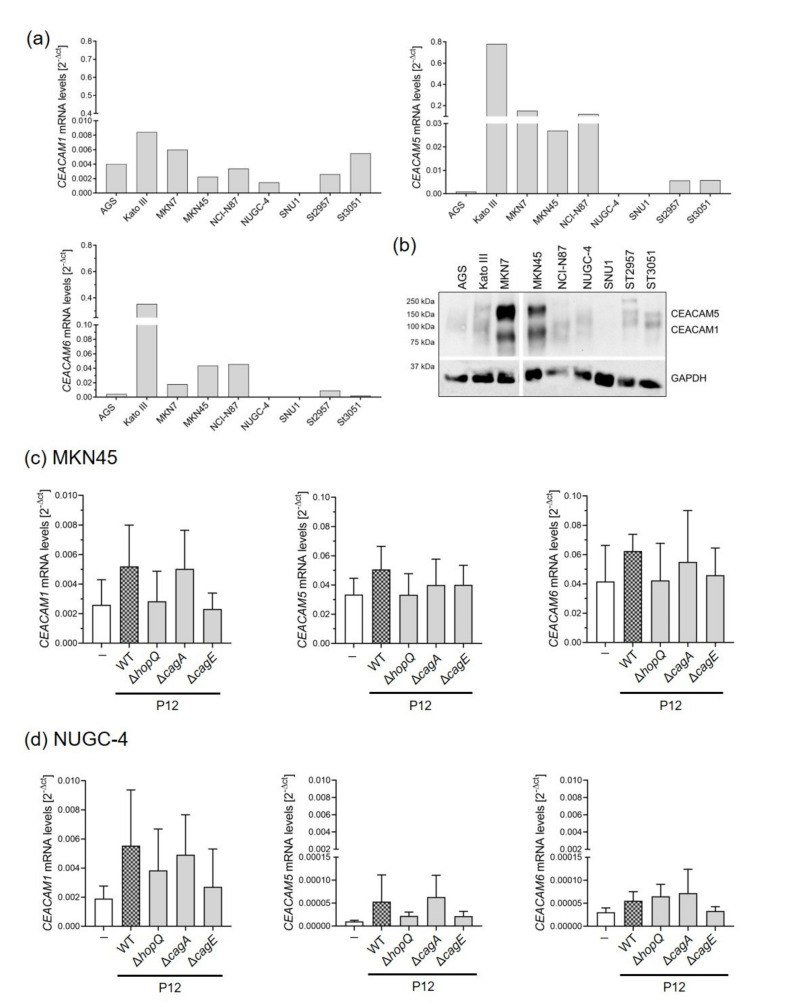
CEACAM expression levels are upregulated upon *H. pylori* infection. Different gastric cancer cells were screened for basal *CEACAM1*, *5* and *6* mRNA (**a**) and protein (**b**) expression by qPCR and Western blot, respectively. MKN45 (**c**) and NUGC-4 (**d**) cells were infected with P12 wild type *H. pylori* strain, or isogenic mutant strains, at an MOI of 10 for 8 h. *CEACAM1*, *5* and *6* mRNA levels were analyzed by qPCR. *GAPDH* was used as a housekeeping gene. All qPCR values were normalized to *GAPDH* and calculated using the 2^−Δct^ equation. Graphs show mean ± SD of at least three independent experiments. One-way ANOVA with Bonferroni correction was used to test differences between means.

After previously finding that HopQ interacts with CEACAM receptors expressed on gastric epithelial cells, we examined whether HopQ-CEACAM binding was required for activation of non-canonical NF-κB. Initially, we screened a panel of gastric cancer cell lines for their basal expression levels of *CEACAM1*, *5* and *6*, which are important for binding of *H. pylori* to the gastric epithelium [19]. We observed that gastric cancer cells showed different CEACAM expression levels at baseline (Figure 2a,b). A few cell lines, such as MKN45 cells, showed expression of all three CEACAMs (1, 5 and 6) (Figure 2a). In contrast, other gastric cells such as NUGC-4, expressed only low levels of CEACAM1, while no CEACAM expression was detected in SNU1 cells at baseline (Figure 2a,b). Interestingly, infection of MKN45 and NUGC-4 cells with wild type *H. pylori* increased *CEACAM* expression levels (Figure 2c,d), while after infection with HopQ mutant strains, no significant changes in *CEACAM* expression were detected (Figure 2c,d). Moreover, *CEACAM5* and *6* mRNA levels were much lower in infected NUGC-4 compared to MKN45 cells after *H. pylori* infection. No *CEACAM* expression was detected in infected SNU1 cells, suggesting that the presence of the ligand (HopQ) favours the expression of the receptor (CEACAM).

### 3.3. HopQ–CEACAM Interaction Is Important for the Activation of the Non-Canonical NF-κB Pathway

**Figure 3:**
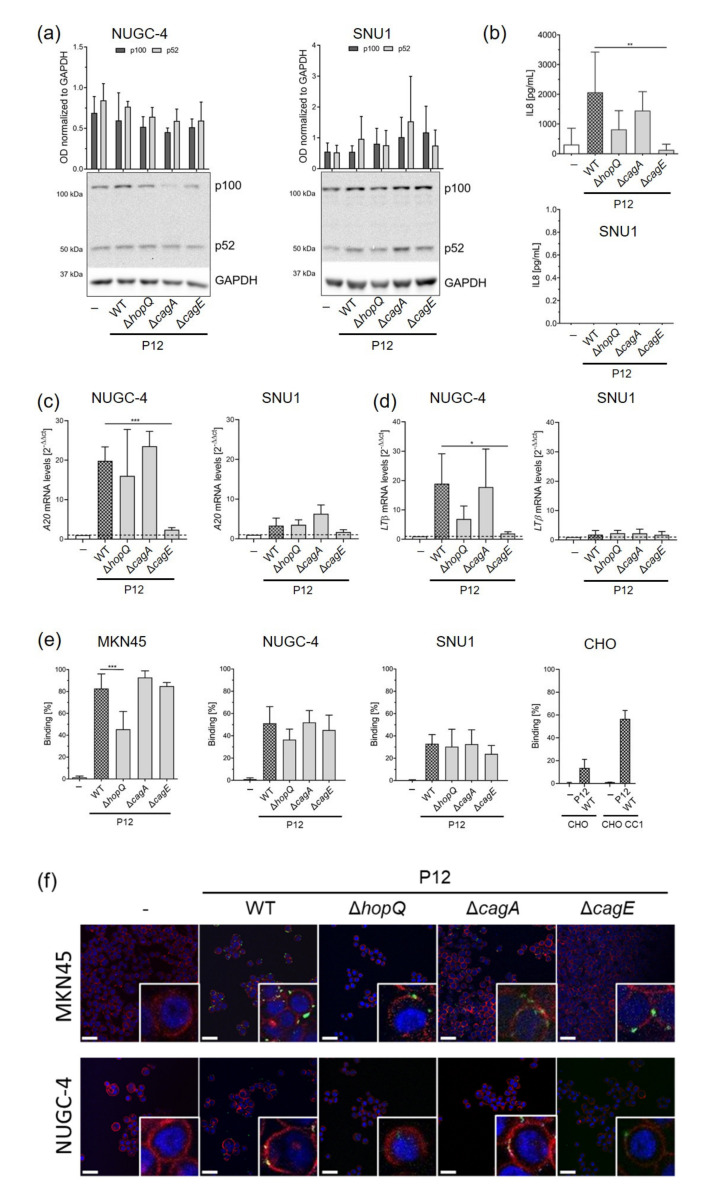
Interaction of HopQ with CEACAM is important for activation of the non-canonical NF-κB pathway. NUGC-4 and SNU1 cells were infected with P12 wild type *H. pylori* strain, or isogenic mutant strains, at an MOI of 10 for 8 h. (**a**) p100 to p52 processing was detected by Western blot. GAPDH served as a loading control and was used for normalization. (**b**) IL8 secretion was measured by ELISA. (**c**) *A20* and (**d**) *LTβ* mRNA levels were analyzed by qPCR. *GAPDH* was used as a housekeeping gene. All qPCR values were normalized to uninfected controls and calculated using the 2^-ΔΔct^ equation. (**e**) Binding assay. CFSE-labeled bacteria were incubated with gastric cancer cells for 30 min with shaking. After washing and fixation, samples were analyzed for GFP signal on a Beckman Coulter Cytoflex flow cytometer. (**f**) Representative confocal images of *H. pylori* P12 wild type, P12Δ*hopQ*, P12Δ*cagA* and P12Δ*cagE* (green) binding to MKN45 and NUGC-4 cells. Nuclei were stained with DAPI (blue), and cell membranes were stained with phalloidin (red). Scale bar: 50 µm. Graphs show mean ± SD of at least three independent experiments. One-way ANOVA with Bonferroni correction was used to test differences between means. * *p* ≤ 0.05; ** *p* ≤ 0.01; *** *p* ≤ 0.001.

Our results from the MKN45 cells suggested that the interaction between HopQ and CEACAM receptors expressed on epithelial cells was necessary for activation of non-canonical NF-κB signalling (Figure 1a). To substantiate this observation and to explore the impact of CEACAM expression on activation of the NF-κB pathway, we infected NUGC-4 cells expressing low levels of CEACAM1 in addition to SNU1 cells, which do not express CEACAMs able to bind to HopQ. Processing of p100 to p52 was not altered in these cell lines upon infection, and no changes between wild type and HopQ deficient bacteria could be detected (Figure 3a).

When analysing IL8 release, lack of HopQ led to a slight decrease in IL8 secretion in infected NUGC-4 cells, while no IL8 secretion was detected in SNU1, regardless the *H. pylori* strain used for infection (Figure 3b).

We next examined activation of canonical NF-κB signalling, by measuring *A20* mRNA expression. A20 is an endogenous inhibitor of the canonical NF-κB pathway and is upregulated upon pathway activation. *A20* levels were increased in NUGC-4 and SNU1 cells upon infection. Lack of HopQ only slightly affected *A20* expression in NUGC-4, while no differences were detected between *H. pylori* wild type and *H. pylori* HopQ-deficient infected cells. Absence of HopQ did not alter *A20* levels in SNU1 cells (Figure 3c).

We also analysed mRNA levels of the non-canonical NF-κB ligand *LTβ*, which was slightly affected by the absence of HopQ in NUGC-4 cells. Again, no changes in *LTβ* expression were detected in SNU1 cells infected with the HopQ deficient strain (Figure 3d).

Finally, we assessed bacterial binding in cells expressing different levels of CEACAMs. We observed that binding of *H. pylori* to MKN45 cells showing high levels of CEACAM expression was highly influenced by the presence of HopQ. In contrast, lack of HopQ barely affected *H. pylori* binding to NUGC-4 cells expressing low CEACAM1 protein levels. Notably, we found very few bacteria bound to SNU1 cells, which lack CEACAM expression (Figure 3e,f).

Taken together, these observations indicate that activation of non-canonical NF-κB is highly dependent on gastric epithelial CEACAM expression levels and thus on bacterial binding to CEACAM through the bacterial protein HopQ.

### 3.4. CEACAM1 Expression and Activation of the Non-Canonical NF-κB Pathway Positively Correlate in Human Gastritis and Gastric Cancer Samples

**Figure 4:**
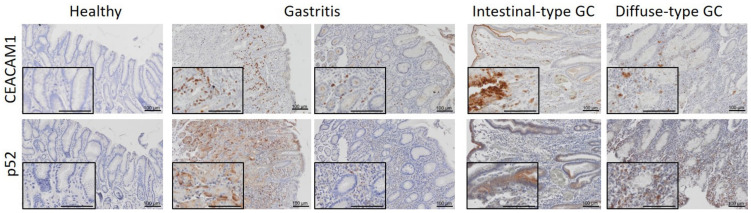
Concomitant CEACAM1 and p52 expression in human gastritis and tumor samples. Representative images of normal mucosa (*n* = 3), gastritis (*n* = 13), intestinal-type gastric cancer (GC) (*n* = 8), and diffuse-type GC (*n* = 3) tissue are shown, which were stained for CEACAM1 and p52 (NF-κB2). Scale bar: 100 µm.

To confirm our previous findings observed in gastric cancer cells in human gastric tissue samples, healthy tissue, gastritis, intestinal-type, and diffuse-type gastric cancer biopsies were stained for CEACAM1 and NF-κB2 by immunohistochemistry (Figure 4). In healthy stomach tissue, no CEACAM1 expression was observed. However, CEACAM1 expression was induced in *H. pylori*-induced gastritis as well as in gastric tumors. Activation of non-canonical NF-κB, as detected by p52 nuclear staining, was observed in *H. pylori*-infected tissue and gastric cancers, correlating with CEACAM1 expression in 46% of patient samples with *H. pylori*-induced gastritis, 62.5% of intestinal-type gastric tumor samples and in 100% of diffuse-type gastric cancer samples analysed.

Taken together, these results indicate that the binding of *H. pylori* to gastric epithelial cells via the HopQ-CEACAM interaction is important for the activation of non-canonical NF-κB in gastric cells.

## 4. Discussion

CEACAM receptors are cell surface proteins expressed in various cell types including epithelial, endothelial and immune cells (e.g., leukocytes, dendritic cells) [45,46]. CEACAMs play an essential role in different biological processes, such as cell adhesion, tumor suppression, angiogenesis or leukocyte activation, and are upregulated in the tissue or in sera during cancer progression in different cancer types such as colorectal or pancreatic cancer [47,48]. Notably, CEACAMs are also surface receptors used by different gram-negative bacteria, such as *Neisseria*, *Eschericia coli*, *Salmonella* as well as by *H. pylori* to bind to the human epithelium [19,46,49]. *H. pylori* possesses different adhesion molecules, each with their own human cell receptor [50]. Binding of *H. pylori* to CEACAM receptors is mediated by the adhesin HopQ. This interaction is not only essential for binding of *H. pylori* to the gastric epithelium but also for cag pathogenicity island (cagPAI)-dependent CagA translocation and induction of IL8 secretion by gastric epithelial cells, thereby modulating the host immune response [19,37,39]. In this immune response, the activation of the non-canonical NF-κB pathway plays a crucial role, as we previously observed that this signalling pathway is important for the recruitment of immune cells to the stomach upon *H. pylori* infection [32]. When assessing the influence of various *H. pylori* virulence factors, including CagA, CagE, BabA, SabA, VacA, gGT or UreA/B on the activation of non-canonical NF-κB pathway, we observed that only a functional T4SS was required [32]. However, the role of bacterial adhesins such as HopQ was not assessed. Considering the previously reported functions of HopQ related to T4SS functionality and downstream signalling, we hypothesised in the current study whether HopQ binding to CEACAM receptors could be important for the activation of the non-canonical NF-κB pathway. To this end, we used gastric cells expressing different levels of CEACAM receptors, as well as cells lacking CEACAMs. This approach allowed us to confirm that HopQ-CEACAM interaction is important for the activation of non-canonical NF-κB.

The involvement of HopQ in T4SS-dependent signalling was firstly reported by Begolova et al., who showed that HopQ is important for the T4SS-dependent activation of canonical NF-κB, the induction of MAPK signalling, and the secretion of IL8 [38]. More recently, Maubach et al. showed similar results [51]. Interestingly, in the latter study, a possible effect of HopQ-CEACAM binding on non-canonical NF-κB was also assessed, and it was concluded that this interaction supports activation of non-canonical NF-κB mediated by the type T4SS [51].

Importantly, we included human gastric tissue samples in our study where we could observe that activation of non-canonical NF-κB relates to expression of CEACAM1. In healthy tissue, no CEACAM1 expression was found but it was upregulated in tissue samples presenting gastric inflammation induced by *H. pylori* as well as in gastric adenocarcinomas, where CEACAM1 may be involved in tumor angiogenesis as reported previously [52,53]. Our in vitro data further suggest that the presence of HopQ is not only important for the activation of non-canonical NF-κB, but also for the induction of the expression of its receptor, because up-regulation of CEACAM expression upon *H. pylori* infection was only observed in cells where HopQ-CEACAM binding occurred. This phenomenon has been described for other pathogens and their corresponding receptors. For instance, in the respiratory tract, non-typeable *Haemophilus influenza* was shown to stimulate the expression of intracellular adhesion molecule 1 (ICAM-1), which in turn functions as its receptor [54,55]. In the gastrointestinal tract, Crohn’s Disease-associated *Escherichia coli* was found to induce the expression of its own receptor, in this case CEACAM6, in ileal epithelial cells [56]. Nevertheless, further research is necessary to determine how HopQ-mediated upregulation of CEACAM expression occurs, paying especial attention to cytokines released and signalling pathways activated after CagA translocation into epithelial cells facilitated by the HopQ-CEACAM interaction.

Different studies support the important role of non-canonical NF-κB signalling in gastric carcinogenesis [32,33,34]. However, despite its involvement in the development and progression of other tumor entities being extensively reported [57,58], further research is necessary to elucidate the molecular mechanisms leading to activation of this pathway in gastric tumors, the consequences of this activation and the identification of possible therapeutic strategies that can be used for targeted treatment. Our current study sheds some light into the mechanisms important for non-canonical NF-κB activation upon *H. pylori* infection and suggests that blocking the HopQ-CEACAM interaction may be a promising strategy to control *H. pylori*-induced gastric malignancies.

## 5. Conclusions

The present study has addressed the question of whether *H. pylori*’s adhesin HopQ is involved in the activation of the non-canonical NF-κB pathway, as this binding protein is important for the functionality of the T4SS. Our data suggest that the HopQ–CEACAM interaction plays an important role in the activation of this pathway by inducing *LTβ* mRNA expression as well as p100 to p52 processing.

## Figures and Tables

**Table 1:** List of human qPCR primers.

Primer	Forward (5′-3′)	Reverse (5′-3′)
*LTβ*	GAG GAC TGG TAA CGG AGA CG	GGG CTG AGA TCT GTT TCT GG
*A20*	TCC TCA GGC TTT GTA TTT GAG C	TCT CCC GTA TCT TCA CAG CTT
*CEACAM1*	GCA ACA GGA CCA CAG TCA AG	CCA GGG CTA CTG CTA TCA G
*CEACAM5*	AGG CCA ATA ACT CAG CCA GT [36]	GGC TTG GGC AGC TCC GC
*CEACAM6*	CGT CGG CAT CAC GAT TGG [37]	TGG GAT TGG AGG AGC TAG AAG [37]
*CCL2*	CTT CGG AGT TTG GGT TTG CTT	CAT TGT GGC CAA GGA GAT CTG
*CXCL10*	TAT TCC TGC AAG CCA ATT TTG TC	TCT TGA TGG CCT TCG ATT CTG
*GAPDH*	GAA GGT GAA GGT CGG AGT	GAA GAT GGT GAT GGG ATT TC

## Data Availability

Not applicable.
